# Effect of GCSB-5, a Herbal Formulation, on Monosodium Iodoacetate-Induced Osteoarthritis in Rats

**DOI:** 10.1155/2012/730907

**Published:** 2012-03-04

**Authors:** Joon-Ki Kim, Sang-Won Park, Jung-Woo Kang, Yu-Jin Kim, Sung Youl Lee, Joonshik Shin, Sangho Lee, Sun-Mee Lee

**Affiliations:** ^1^School of Pharmacy, Sungkyunkwan University, Suwon, Gyeonggi-do 440-746, Republic of Korea; ^2^Business Development, Green Cross Corporation, Yongin, Gyeonggi-do 446-770, Republic of Korea; ^3^Jaseng Hospital, 635 Sinsa-Dong, Gangnam-Gu, Seoul, Republic of Korea

## Abstract

Therapeutic effects of GCSB-5 on osteoarthritis were measured by the amount of glycosaminoglycan in rabbit articular cartilage explants *in vitro*, in experimental osteoarthritis induced by intra-articular injection of monoiodoacetate in rats *in vivo*. GCSB-5 was orally administered for 28 days. *In vitro*, GCSB-5 inhibited proteoglycan degradation. GCSB-5 significantly suppressed the histological changes in monoiodoacetate-induced osteoarthritis. Matrix metalloproteinase (MMP) activity, as well as, the levels of serum tumor necrosis factor-*α*, cyclooxygenase-2, inducible nitric oxide synthase protein, and mRNA expressions were attenuated by GCSB-5, whereas the level of interleukin-10 was potentiated. By GCSB-5, the level of nuclear factor-*κ*B p65 protein expression was significantly attenuated but, on the other hand, the level of inhibitor of *κ*B-*α* protein expression was increased. These results indicate that GCSB-5 is a potential therapeutic agent for the protection of articular cartilage against progression of osteoarthritis through inhibition of MMPs activity, inflammatory mediators, and NF-*κ*B activation.

## 1. Introduction

Osteoarthritis (OA) is a degenerative joint disease characterized by joint pain and a progressive loss of articular cartilage. It has been suggested that biochemical alterations occur within the articular cartilage resulted in imbalance between synthetic and degradative pathways [1]. A key step in the pathophysiology of OA is breakdown of extracellular matrix of articular cartilage by tissue proteinases, enzymes whose expression is upregulated by inflammatory stimuli, such as primary inflammatory cytokines [2]. Nonsteroidal anti-inflammatory drugs (NSAIDs) are effective in the management of OA inflammation. However, the adverse events secondary to NSAIDs was focused on upper gastrointestinal tolerability [3]. In recent years, gene therapy targeted at cytokines offers new hope to OA treatment, and the current focus is on the use of biological agents that block the activity of inflammatory cytokines [4]. Since there are many proinflammatory cytokines, oxidants and other factors exerting action in initiation and development of OA, it is hard to obtain complete therapeutic effects by blocking the activity of one or two cytokines. Developing therapeutics from herbal sources may reduce the risk of toxicity or adverse effects when the drug is clinically used [5] and may exert strong, multifunctional anti-inflammatory effect like many natural products do. Therefore, efforts are being made to elucidate the role of natural products for the treatment of OA.

 GCSB-5 is a purified extract from a mixture of 6 oriental herbs which are the ingredients of Chung-Pa-Juhn used in Jaseng Hospital (Seoul, Korea) and that have been used in traditional medicine to treat inflammatory diseases and bone disorders. Ledebouriellae Radix is reported to have anti-inflammatory effects on Freund's adjuvant-induced arthritis in rats [6]. Cimifugin, a major active component of Ledebouriellae Radix, exhibits inhibitory effects on the synthesis of NO induced by LPS in macrophage cell line RAW 264.7 [7]. Achyranthis Radix shows anti-inflammatory property and inhibits free radicals, such as ONOO^−^, HOCl, and OH radical [8]. 20-Hydroxyecdysone, which is a major active compound of Achyranthis Radix, has beneficial effects on joint and bone in ovariectomized rats [9]. Acanthopanacis Cortex is known to show antiarthritic activity [10], and Cibotii Rhizoma is known for its analgesic property [11] along with osteoclast formation inhibition [12]. Glycine Semen is effective in reducing swelling [13] and genistin, an active compound from Glycine Semen, shows beneficial effect on bone loss [14]. Eucommiae Cortex exhibits strong analgesic effect [15] and geniposide from its extract shows anti-inflammatory effect on rheumatoid arthritis rats [16] and enhances the osteoblast-like cell proliferation and inhibited osteoclast [17]. We reported strong antinociceptive and anti-inflammatory properties of GCSB-5 [11, 13]. Recently, GCSB-5 reduces the development of acute and chronic inflammation, and its anti-inflammatory property is likely due to inhibition of inducible nitric oxide synthase (iNOS) and cyclooxygenase (COX)-2 expression via downregulation of the Akt signal pathway and inhibition of nuclear factor-**κ**B (NF-**κ**B) activation [18]. In phase III clinical study, GCSB-5 was shown to exert therapeutic effects and acted to reduce OA severity and improved functional recovery without apparent hepatic or renal toxicity (unpublished data).

 In this study, we examined the chondroprotective and anti-inflammatory effects of GCSB-5 on monoiodoacetate (MIA)-induced OA animal model, both *in vitro* and *in vivo*.

## 2. Materials and Methods

### 2.1. Preparation and Composition of GCSB-5

 GCSB-5 was prepared by the Hanpoong Pharmaceutical Co., Ltd., Jeonju, Republic of Korea. The mixture of six crude drugs (Ledebouriellae Radix (4.444 g), Achyranthis Radix (4.444 g), Acanthopanacis Cortex (4.444 g), Cibotii Rhizoma (2.778 g), Glycine Semen (2.778 g), and Eucommiae Cortex (1.389 g)) was powdered and boiled for 3 h in distilled water (1 L). The resulting extract was subjected to ultrafiltration, and the components with molecular weight over 10,000 were excluded. The filtrate was lyophilized as powder and kept at 4°C until use. GCSB-5 was administered orally at a dose of 300 and 600 mg/kg in saline (1 kg/10 mL), and the same volume of saline was used as a vehicle control group. The validation of GSCB-5 was performed by high-performance liquid chromatography analysis of each ingredient extract using six indicator biological components: cimifugin for Ledebouriellae Radix, 20-hydroxyecdysone (0.311-0.312 mg/g) for Achyranthis Radix, acanthoside D (0.577-0.578 mg/g) for Acanthopanacis Cortex, onitin-4-O-*β*-D-glucopyranoside for Cibotii Rhizoma, genistin (0.0426-0.0427 mg/g) for Glycine Semen, and geniposide (0.431-0.432 mg/g) for Eucommiae Cortex. GCSB-5 was further standardized for quality control according to the regulations imposed by Korea Food and Drug Administration (KFDA).

### 2.2. Chemicals

 Dulbecco's modified Eagle's medium (DMEM), penicillin/streptomycin (10,000 U/mL, 10,000 *μ*g/mL, resp.), and fetal bovine serum (FBS) were obtained from Gibco BRL, Life Technologies (Grand Island, NY, USA). All the other materials required for culturing of tissue were purchased from Sigma Chemical Company (St. Louis, MO, USA).

### 2.3. Animals

 Male Sprague-Dawley rats (200–220 g) and male New Zealand white rabbits (2.0–2.2 kg) were obtained from Dae Han Biolink Ltd. (Eumseong, Korea) and housed in solid bottom cages with pellet food and water available *ad libitum*. All animal procedures were approved by the Sungkyunkwan University Animal Care Committee and were performed in accordance with the guidelines of the National Institutes of Health.

### 2.4. Cartilage Glycosaminoglycan Assay

 Rabbit knee articular cartilage explants were obtained according to the method described by Sandy et al. [19]. Briefly, 200–220 mg articular surfaces per joint were dissected and submerged into complete medium of DMEM supplemented with heat-inactivated 5% FBS, penicillin (100 U/mL), and streptomycin (100 *μ*g/mL). After stabilization in incubator, the medium was replaced with basal medium made of DMEM supplemented with heat-inactivated 1% FBS, 10 mM HEPES, penicillin (100 U/mL), and streptomycin (100 *μ*g/mL). Cartilage pieces (50–60 mg; 2 × 3 × 0.35 mm/piece) were placed in 24-well cell culture plates and treated with GCSB-5 at 1 × 10^−3^, 1 × 10^−2^, and 1 × 10^−1^ mg/mL or 30 *μ*M diclofenac (Sigma-Aldrich, St. Louis, MO, USA). After 1 h of GCSB-5 or diclofenac pretreatment, 5 ng/mL of rhIL-1**α** (R&D Systems, Minneapolis, MN, USA) was added and further incubated at 37°C in a humidified 5% CO_2_/95% air incubator. The amount of glycosaminoglycan (GAG) in the medium was determined by the 1,9-dimethyl-methylene blue method using the Blyscan Sulfated GAG Assay kit (Biocolor Ltd., County Antrim, UK) according to the manufacturer's instructions.

### 2.5. MIA-Induced OA

 Rats were anesthetized with diethyl ether and given a single intra-articular injection of 3 mg MIA (Sigma-Aldrich, St. Louis, MO, USA) through the infrapatellar ligament of the left knee [20]. MIA was dissolved in physiological saline and administered in a 50 *μ*L volume. Rats were treated with saline, with 300 or 600 mg/kg of GCSB-5 or with 5 mg/kg of diclofenac by oral administration once daily, for 2, 7, and 28 days since MIA injection. These GCSB-5 doses and MIA injection volume were selected based on previous evaluations [21].

### 2.6. Gross Observation

 After MIA injection, all experimental rats were weighed and carefully inspected every 2 days to assess knee joint swelling and gait disturbances under natural conditions in the cages, where they moved freely. Swelling and limping were classified as no change, mild, and severe on the basis of severity [22], and inspection was conducted by an inspector blinded to treatment details throughout the study.

### 2.7. Roentgenographic Examination and Histopathological Analysis

 Seven and 28 days following MIA injection, rats were checked with roentgenography to assess chronic morphological changes of knee articular bones for narrowing, loss of joint region, cartilage erosion, and osteophyte formation [23]. For histological analysis, knee joints were removed and fixed in 10% neutral buffered formalin, decalcified with 10% formic acid, and embedded in paraffin. Five micrometer (5 *μ*m) sections were stained with hematoxylin and eosin (H and E) or safranin-O fast green (SOFG) and observed. Histopathological changes in each animal were quantitatively expressed by three grades for each finding [24]. Grading was done under the authority of Medplan Pathology Laboratories, Seoul, Korea.

### 2.8. Gelatinase Assay

 Rat articular cartilage samples of MIA-induced OA were harvested 7 and 28 days after MIA injection. Gelatinase activities were measured by the gelatin zymography method described by Dumond et al. [25]. Proteins were extracted from pulverized cartilage tissues and electrophoresed on 10% zymogram precast gels. The cleared gels were captured, and the area of each band was quantified with densitometric scanning analysis program (Science Lab 98 Image Gauge, version 3.12, Fuji Photo Film Co. Ltd., Tokyo, Japan).

### 2.9. Serum Cytokine Levels

Commercial tumor necrosis factor (TNF)-**α**, interleukin (IL)-1**β*, *and IL-10 enzyme-linked immunosorbent assay (ELISA) kits (BD Biosciences Co., CA, USA) were used for quantification of the serum levels of TNF-**α**, IL-1**β*,* and IL-10, respectively.

### 2.10. Western Blot Immunoassay

 15 *μ*g of whole protein was used for determination of the content of COX-2 and iNOS. 20 *μ*g of nuclear protein was used for determination of the content of the NF-**κ**B/p65 subunit. 20 *μ*g of the cytosolic protein was used for determination of the content of the inhibitor of **κ**B (I**κ**B)-**α**. ImageQuantTM TL software (Amersham Biosciences/GE Healthcare, Piscataway, NJ, USA) was used for densitometric evaluation of visualized immunoreactive bands. The following primary antibodies were used: COX-2 (Abcam, Cambridge, UK; 1 : 1000), iNOS (Transduction Lab., CA, USA; 1 : 1000), phosphoryl NF-**κ**B/p65 (Santa Cruz Biotechnology, Santa Cruz, CA, USA; 1 : 1000), and I**κ**B-**α** (Santa Cruz Biotechnology; 1 : 5000) were used, and the signals were normalized to that of **β**-actin (Sigma Chemical Co.; 1 : 1000) or lamin B1 (Abcam; 1 : 2500).

### 2.11. Total RNA Extraction and Reverse Transcription-Polymerase Chain Reaction (RT-PCR)

 Articular cartilage samples collected 2 and 28 days after MIA injection were pulverized in TRI Reagent (Molecular Research Center Inc., Cincinnati, OH, USA) for RNA extraction. Equal amounts of RNA from articular cartilages were subjected to reverse transcription using iNtRON RNA PCR kit (iNtRON Biotechnology Co., Seongnam, Korea) to generate cDNA for RT-PCR analysis. RT-PCR analysis was performed with the GeneAmp PCR system 2700 (Applied Biosystems Co., Foster City, CA, USA). The primers used in the RT-PCR are listed in Table 1. All PCR reactions included an initial denaturation step at 94°C for 5 min and a final extension at 72°C for 7 min. The PCR amplification cycling conditions were as follows: 32 cycles of 94°C (30 s), 58°C (30 s), and 72°C (30 s) for *TNF-*α**; 32 cycles of 94°C (45 s), 65°C (45 s), and 72°C (60 s) for *iNOS*; 40 cycles of 94°C (45 s), 65°C (45 s), and 73°C (60 s) for *COX-2*; 36 cycles of 94°C (30 s), 60°C (30 s), and 72°C (45 s) for *IL-1*β**; 40 cycles of 94°C (30 s), 66°C (45 s), and 72°C (45 s) for *IL-10*; 30 cycles of 94°C (30 s), 56°C (30 s), and 72°C (60 s) for **β*-actin*. After RT-PCR, 10 *μ*L samples of the amplified products were resolved by electrophoresis on 1.5% agarose gels and stained with ethidium bromide. The intensity of each PCR product was evaluated semiquantitatively using a digital camera (DC120; Eastman Kodak, Rochester, NY, USA) and a densitometric scanning analysis program (ID Main; Advanced American Biotechnology, Fullerton, CA, USA).

### 2.12. Statistics

 All results are presented as mean ± S.E.M. The overall significance of the experimental results was examined by one-way analysis of variance and the two-tail Dunnet's *t*-test. Differences between groups were considered significant at *P* < 0.05 with the appropriate Bonferroni correction for multiple comparisons.

## 3. Results

### 3.1. Cartilage Glycosaminoglycan Release

**Figure 1:**
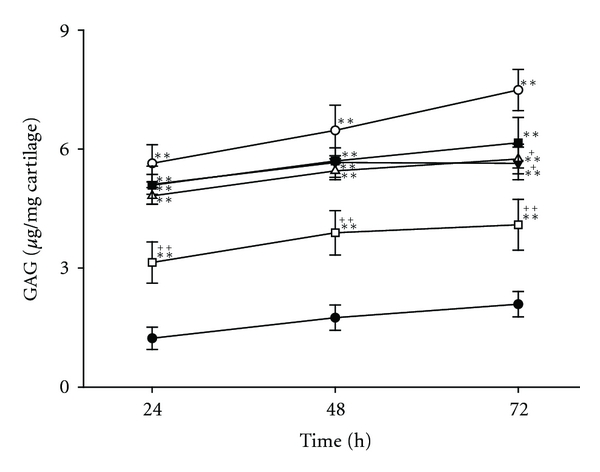
GAG release in rabbit articular cartilage explant cultures at 24, 48, and 72 h. Rabbit articular cartilage explants were stimulated with rhIL-1**α** (5 ng/mL). The amount of GAG release stimulated by rhIL-1**α** (∘) increased approximately 3.6 times compared to control (●) at 72 h. GCSB-5 (1.0 × 10^−3^ (*▼*) and 1.0 × 10^−2^ (∆) mg/mL) and diclofenac (30 *μ*M (□)) efficiently inhibited the GAG release. However, a high concentration of GCSB-5 (1.0 × 10^−1^ (■) mg/mL) slightly inhibited it. Each value represents the mean ± S.E.M. from 6 articular cartilage explants cultures per group. **Significantly different (*P* < 0.01) from control. ^+^ and ^++^Significantly different (*P* < 0.05, *P* < 0.01) from rhIL-1**α**.

 In the control group, the level of GAG in the culture medium remained constant at approximately 1.5 *μ*g/mg cartilage throughout the experiment. In the rhIL-1**α**-treated group, on the other hand, the level of GAG in the culture medium dramatically increased to approximately 4 times the control values. GCSB-51 × 10^−3^ and 1 × 10^−2^ mg/mL treatments attenuated the elevation in GAG release at 72 h (Figure 1).

### 3.2. Gross Observation

 In the MIA-injected groups, swelling and limping were first observed 7 days after MIA injection. They subsided transiently and then reappeared at 14 days. These symptoms gradually aggravated at 21 days (data not shown) and were the most severe at 28 days. Twenty-eight days after MIA injection, swelling and limping were attenuated by both 300 and 600 mg/kg GCSB-5 treatment (Table 2).

### 3.3. Roentgenographic and Histopathological Analysis

**Figure 2:**
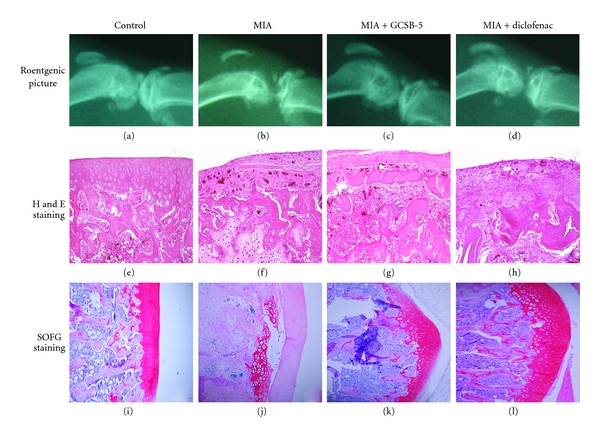
Roentgenography and histopathological features of osteoarthritic lesion in the knee joint of rats 28 days after intra-articular injection of MIA (H and E staining, ×100; SOFG staining, ×100). Control (a) represents intact normal joint feature. Vehicle-treated MIA (b) shows a severely damaged joint with rough edges around the tibia and femur, indicative of bone lysis, swelling, and tendency of patellar displacement. However, these damages were reduced significantly by treatment with 600 mg/kg GCSB-5 (c) and 5 mg/kg diclofenac (d). SOGF-stained control (e) represents normal cartilage PG staining, whereas vehicle-treated MIA (f) represents severely damaged cartilage showing marked fibrillation and the depletion of SOFG staining with separation of cartilage from subchondral bone. 600 mg/kg GCSB-5 (g) and 5 mg/kg diclofenac (h) treatments significantly reduced cartilage damage. H and E stained control (i) represents the normal status of joint cartilage, whereas vehicle-treated MIA (j) represents severely damaged cartilage showing widespread cell necrosis and inflammation. However, treatment with 600 mg/kg GCSB-5 (k) and 5 mg/kg diclofenac (l) treatments significantly reduced joint cartilage damage.

Seven days after MIA injection, rats underwent the first roentgenographic examination. Their roentgenographic examinations revealed degenerative changes, such as irregularity or osteophytes on the surface of the cartilage and subchondral bone (data not shown). At 28 days, rats underwent the second roentgenographic examination (Figures 2(a)–2(d)). Morphological changes were more significant, showing rough edges of cartilage and the tendency of patellar displacement. These changes were attenuated by GCSB-5 600 mg/kg treatment. Twenty-eight days after MIA injection, H and E staining revealed irregular surface accompanied by ulceration, fibrillation, and loss of cartilage tissue (Figures 2(e)–2(h)). However, these cartilage damages were attenuated by GCSB-5 600 mg/kg treatment. SOFG staining also revealed clearly diffused PG depletion in joint cartilage tissues of MIA-injected rats (Figures 2(i)–2(l)). This loss of PG was attenuated by GCSB-5 600 mg/kg treatment. Summation of all histopathologic finding scores in vehicle-treated MIA group and in 300 and 600 mg/kg GCSB-5-treated MIA groups were 24.5 ± 1.3, 16.1 ± 1.4, and 12.5 ± 1.1, respectively (Table 3).

### 3.4. Gelatinase Assay

**Figure 3:**
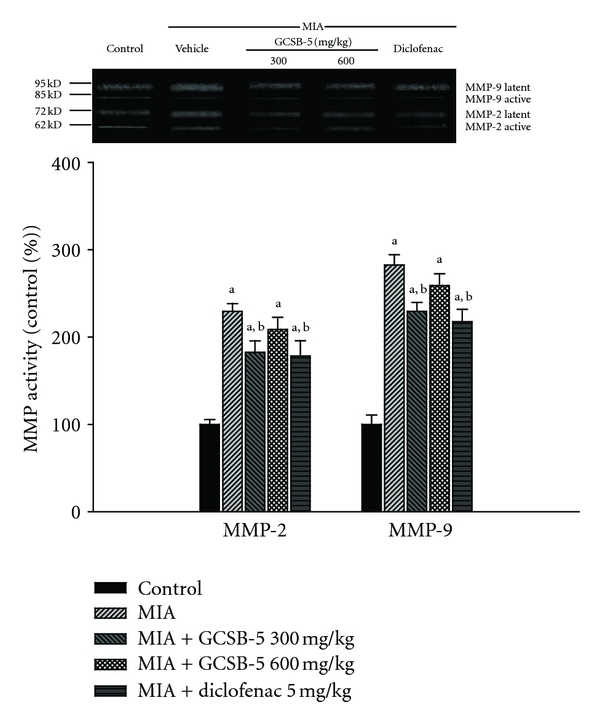
Activities of MMP-2 (Gelatinase A) and MMP-9 (Gelatinase B) assessed by zymography in knee joint cartilages obtained 28 days after MIA injection. The latent and active amounts of gelatinase were combined to give a total value for each gelatinase. Each value represents the mean ± S.E.M. from 6 rats per group. ^a^Significantly different (*P* < 0.01) from control. ^b^Significantly different (*P* < 0.05) from vehicle-treated MIA.

 Seven days after MIA injection, the activities of matrix metalloproteinase (MMP)-2 and -9 increased to 2.7 and 2.4 times that in the control group, respectively. Similarly, 28 days after MIA injection, the activities of MMP-2 and -9 increased to 2.3- and 2.8-fold higher than the control level, respectively. On day 7, GCSB-5 and diclofenac treatment showed no significant modulation on MMP activities (data not shown). However, on day 28, GCSB-5 300 mg/kg treatment exhibited significant MMP-2 and -9 activities attenuation (79.6%, *P* < 0.01 and 81.2%, *P* < 0.01, resp.), while GCSB-5 600 mg/kg treatment did not affect the MMP-2 and -9 activities (91.0% and 91.6%, resp.) (Figure 3).

### 3.5. Inflammatory Mediators

 The serum levels of TNF-**α**, IL-1**β*,* and IL-10 were 30.0 ± 4.5 pg/mL, 29.1 ± 3.7 pg/mL, and 27.8 ± 0.6 pg/mL in the control. 2 days after MIA injection, the serum levels of TNF-**α**, IL-1**β*,* and IL-10 increased to 2.8-, 3.4- and 2.2-fold higher than the control level, respectively. Increase in TNF-**α** level was significantly suppressed by treatment with GCSB-5, while increase in IL-10 level was significantly potentiated by GCSB-5. However, GCSB-5 did not affect the serum level of IL-1**β** (Table 4). The levels of COX-2 and iNOS protein expression increased 3.3 and 12 times in the vehicle-treated MIA groups, compared to those in the control group 2 days after MIA injection, respectively (Figure 5). Increase in COX-2 and iNOS protein expression was significantly suppressed by treatment with GCSB-5.

**Figure 4:**
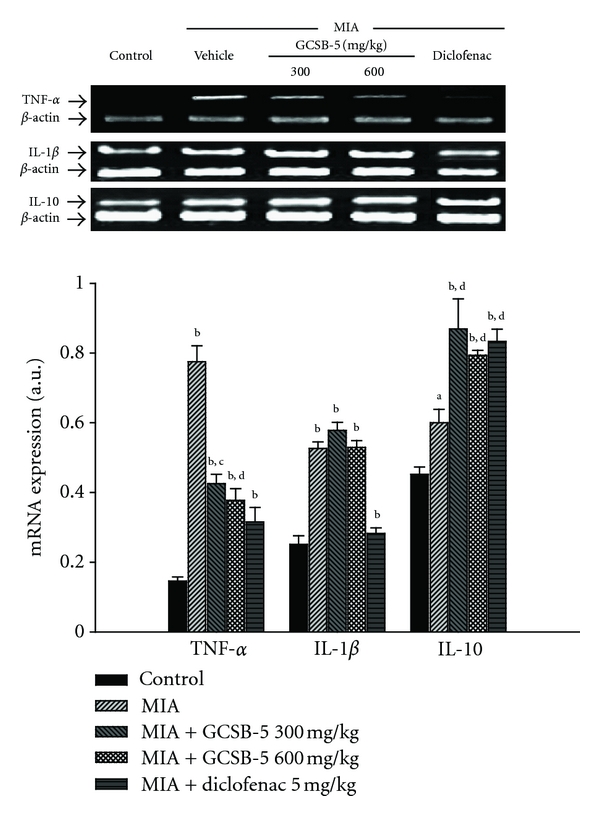
*TNF-*α**, *IL-1*β**, and *IL-10* mRNA expressions in cartilage from knee joints of rats at day 2 after MIA injection. Each value represents the mean ± S.E.M. from 6 rats per group. ^a,b^Significantly different (*P* < 0.01, *P* < 0.05) from control. ^c,d^Significantly different (*P* < 0.01, *P* < 0.05) from vehicle-treated MIA.

**Figure 5:**
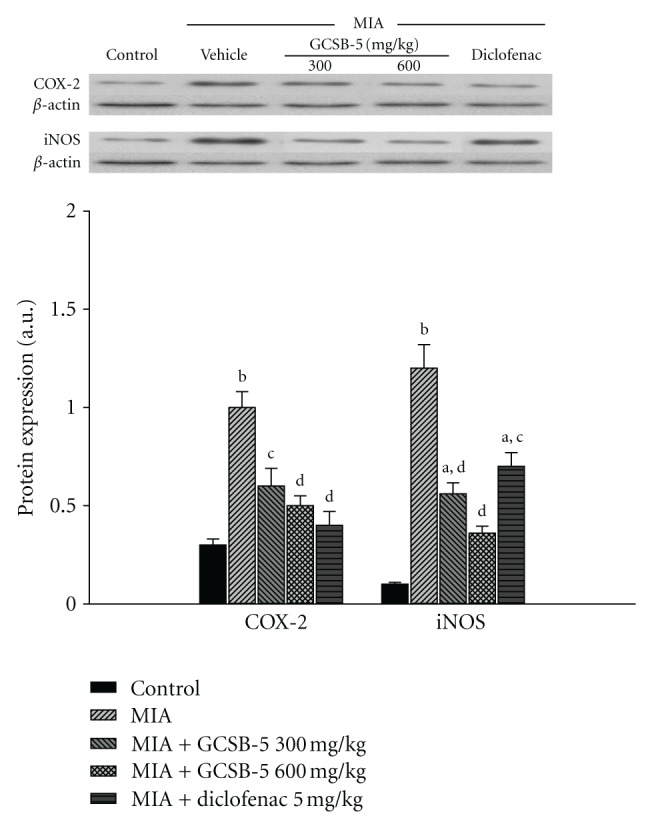
COX-2 and iNOS protein expressions in cartilage from knee joints of rats at day 2 after MIA injection. Each value represents the mean ± S.E.M. from 6 rats per group. ^a,b^Significantly different (*P* < 0.01, *P* < 0.05) from control. ^c,d^Significantly different (*P* < 0.01, *P* < 0.05) from vehicle-treated MIA.

**Figure 6:**
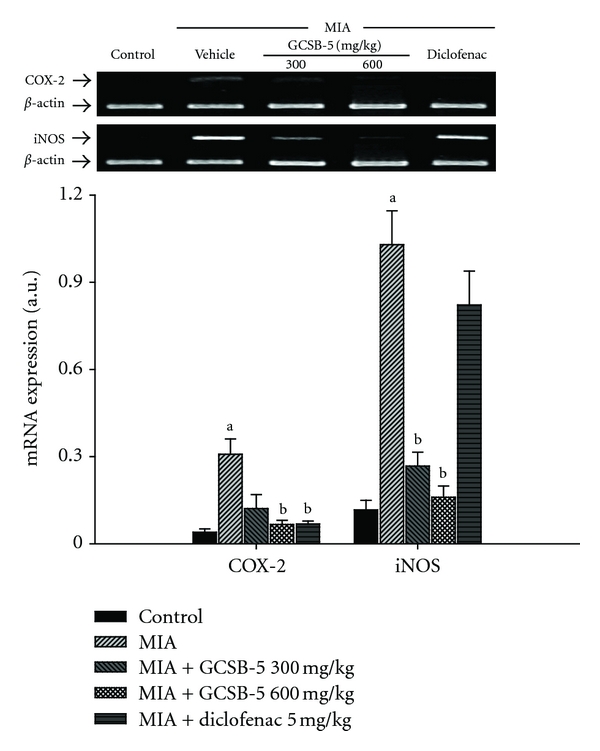
*COX-2* and *iNOS* mRNA expressions in cartilage from knee joints of rats at day 2 after MIA injection. Each value represents the mean ± S.E.M. from 6 rats per group. ^a^Significantly different (*P* < 0.05) from control. ^b^Significantly different (*P* < 0.05) from vehicle-treated MIA.

 The levels of *TNF-*α**, *IL-1*β**, *IL-10*, *COX-2,* and *iNOS* mRNA expression increased 5.3, 2.1, 1.3, 7.8 and 8.8 times in the vehicle-treated MIA groups, compared to those in the control group 2 days after MIA injection, respectively (Figures 4 and 6). Increase in *TNF-*α**, *COX-2,* and *iNOS* mRNA expression was significantly suppressed by treatment with GCSB-5. However, GCSB-5 did not affect the level of *IL-1*β** mRNA expression. Interestingly, increase of *IL-10* mRNA expression was significantly potentiated by GCSB-5. At 7 and 28 days, there were no significant differences in the level of inflammatory mediators mRNA expression among any of the experimental groups (data not shown).

### 3.6. Nuclear NF-*κ*B and Cytosolic I*κ*B-*α* Immunoblot Assay

**Figure 7:**
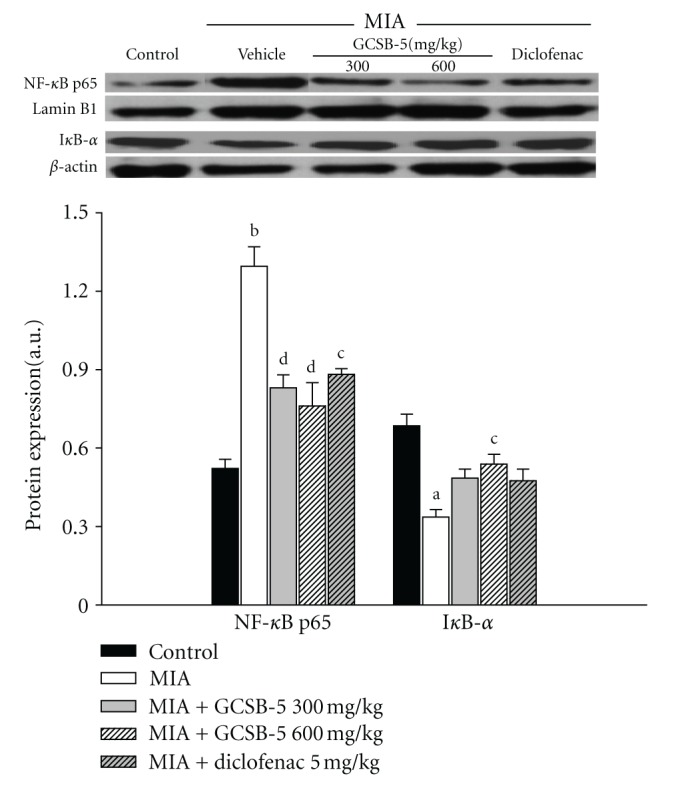
Nuclear NF-**κ**B p65 and cytosolic I**κ**B-**α** protein expressions in cartilage from knee joints of rats at day 2 after MIA injection. Each value represents the mean ± S.E.M. from 6 rats per group. ^a,b^Significantly different (*P* < 0.05, *P* < 0.01) from control. ^c,d^Significantly different (*P* < 0.05, *P* < 0.01) from vehicle-treated MIA.

 The nuclear localization of NF-**κ**B was measured by the protein level of NF-**κ**B p65 subunit in the nucleus. Cytosolic I**κ**B-**α** was also examined from cytosol fraction as an endogenous NF-**κ**B inhibitor. The level of nuclear NF-**κ**B p65 protein expression increased 2.5 times, whereas the level of cytosolic I**κ**B-**α** protein expression decreased 2.0-fold in the vehicle-treated MIA group, compared to that in the control group 2 days after MIA injection. These changes were significantly inhibited by GCSB-5 treatment (Figure 7).

## 4. Discussion

 Today, cure for OA remains elusive. The management of OA is largely palliative focusing on the alleviation of symptoms. Current recommendations for the management of OA include a combination of nonpharmacological (weight loss, education programs, and exercise) and pharmacological interventions (paracetamol, NSAIDs, etc.) [26]. Of the pharmacological intervention available, analgesics and NSAIDs have been proven to be highly effective in controlling the symptoms and signs of OA. However, they have potential gastrointestinal (GI) adverse effects. Herbal medicinal products (HMPs) are not yet among the recommended treatment options, although they are used in a variety of oral and topical forms in the treatment of OA. The mechanism of action of HMPs is broader than that of NSAIDs and/or analgesics in current use for symptomatic OA. Although the exact mechanisms of action have not yet been elucidated, there is no doubt that all herbal medicines act via several pathways, including inhibition of COX and/or lipoxygenase, inhibition of cytokine release, inhibition of elastase or hyaluronidase, and induce antioxidative activity [27]. On the basis of this knowledge, our experimental herbal extract, GCSB-5, consisted of various herbs known to exhibit antiarthritic, anti-inflammatory and analgesic effects, is expected to show therapeutic activity against OA.

 Articular cartilage destruction is a key pathological characteristic of OA. MIA is an inhibitor of glyceraldehyde-3-phosphate dehydrogenase activity, and therefore an inhibitor of glycolysis shown to induce chondrocyte death *in vitro *[28]. Intra-articular injection of MIA induces chondrocyte death in the articular cartilage of rodent and nonrodent species [29]. Injection of MIA into the knees of rats provides a model in which lesions resembling some aspects of human OA produced quickly and has been suggested as a model for the study of chondroprotective drugs [30]. In the present study, we investigated GCSB-5 on the clinical and behavioral changes associated with MIA-induced OA. Swelling and limping were apparent as early as 7 days after MIA injection, after which they became transiently subsided. At 14 days, there was a second period of knee joint swelling and limping that was progressively aggravated until day 28. Administration of GCSB-5 once daily for 28 days significantly reduced the severity of swelling and limping. These results suggest that GCSB-5 may have potential as a treatment for OA. Roentgenographic and histological observations strongly supported the behavioral changes following MIA injection as well as the protective effect of GCSB-5.

 Cartilage comprises an extracellular matrix consisting of PGs, collagens (types II, IX, XI, and others), and water. Cartilage PGs consist of a protein core with GAG side chains [31]. When cartilage is damaged by inflammatory mediators such as rhIL-1**α**, PGs degrade and consequently release GAG, which is a typical clinical symptom of OA. GCSB-5 did not inhibit GAG release at low concentrations, but showed an inhibitory effect at moderate-to-high concentrations. This analysis reflected the histochemical appearance of cartilage. SOFG staining showed significant PG loss and lesion development in subchondral bone which were induced by MIA injection. These cartilage damages were attenuated by GCSB-5 treatment. Our data suggest that GCSB-5 may protect articular cartilage from degradation.

 MMPs are a family of proteinases that together can degrade all extracellular matrix components. Type IV collagenases (gelatinases) are members of the MMP family and are thought to play an important role in the degradation of extracellular components. The gelatinase subclass can be divided into gelatinase-A (MMP-2) and gelatinase-B (MMP-9), which is capable of degrading types IV and V collagens, elastin, and gelatin [32]. MMP-2 is known to be produced by osteoblasts and tissue structural cells, including fibroblasts and endothelial cells, whereas MMP-9 is produced by inflammatory cells such as macrophages, neutrophils, and eosinophils [33, 34]. These MMPs are secreted as latent precursors and can be activated by limited proteolysis. The increased expression of MMP-2 and -9 in the synovium of patients with arthritic effusions superiorly reflects the inflammatory condition of the joints, and a positive correlation between MMP-9 production and rapid destruction of the hip joint has been described in OA [35]. Although GCSB-5 did not affect MMP-2 and -9 activities 7 days after MIA injection (data not shown), GCSB-5 especially at a dose of 300 mg/kg suppressed MMP-2 and -9 activities 28 days after MIA injection. Our results indicate that GCSB-5 inhibits collagen degradation through inhibition of MMP-2 and -9 activities in late stage of OA.

 Matrix turnover is solely dependent on chondrocytes, which are believed to be the main site of inflammatory mediators production in human OA [36]. Overexpression of MMPs is induced by several cytokines, such as TNF-**α**, IL-1, IL-17, and IL-10. TNF-**α** and IL-1**β** drive the catabolic processes in OA, leading to cartilage degradation. In this study, the levels of *TNF-*α**, *COX-2,* and *iNOS* mRNA expression were significantly increased 2 days after MIA injection and returned to control level at 28 days (data not shown). These increases were attenuated by GCSB-5 treatment. On the other hand, a critical function of IL-10 is to limit inflammatory responses [37]. This cytokine inhibits IL-1**β** and TNF-**α** expression and is present in OA chondrocytes, where it may counteract their catabolic effects [38]. Interestingly, GCSB-5 treatment significantly potentiated this increase. Our results indicate that GCSB-5 shows a significant anti-inflammatory action in the early stage of OA.

 Inappropriate regulation of NF-**κ**B activity has been implicated in the pathogenesis of inflammatory diseases, such as rheumatoid arthritis and OA [39]. NF-**κ**B signaling pathways mediate critical events in the inflammatory response by chondrocytes, leading to progressive extracellular matrix damage and cartilage destruction. NF-**κ**B mediates fibronectin fragment-induced chondrocyte activation and increased expression of proinflammatory cytokines, chemokines as well as MMPs such as IL-6, IL-8, MCP-1, growth-related oncogene-**α**, -**β**, -**γ*,* and MMP-13 by human articular chondrocytes [40, 41]. In this study, we showed that GCSB-5 inhibits nuclear translocation of NF-**κ**B/p65 subunit and degradation of I**κ**B-**α**.

Although the results of present study provided clues for further studies on the pharmacological mechanisms of GCSB-5, the relationship between the effects and its active components remains to be clarified. Therefore, the detailed molecular mechanisms of GCSB-5 and further studies of anti-inflammatory properties of the active ingredients should be elucidated.

## 5. Conclusions

 These results indicate that GCSB-5 improves OA-induced cartilage damage, which inhibits MMP activities, downregulates the expression of inflammatory mediators, and suppresses NF-**κ**B activity, suggesting that GCSB-5 may be a potential therapeutic agent for OA.

## Figures and Tables

**Table 1:** RT-PCR primers used in study.

Gene	Accession number	Primer sequences (5′ → 3′)	Product length (bp)
*TNF-*α**	X66539	Sense: GTA GCC CAC GTC GTA GCA AA	346
		Antisense: CCC TTC TCC AGC TGG AAG AC	
*iNOS*	D44591	Sense: TTC TTT GCT TCT GTG CTT AAT GCG	1061
		Antisense: GTT GTT GCT GAA CTT CCA ATC GT	
*COX-2*	U03389	Sense: CTG CAT GTG GCT GAT GTC ATC	1061
		Antisense: AGG ACC CGT CAT CTC CAG GGT AAT C	
*IL-1*β**	M98820	Sense: TGA TGT TCC CAT TAG ACA GC	378
		Antisense: GAG GTG CTG ATG TAC CAG TT	
*IL-10*	X60675	Sense: CAG TCA GCC AGA CCC ACA T	322
		Antisense: GCT CCA CTG CCT TGC TTT	
**β*-Actin*	V01217	Sense: TTG TAA CCA ACT GGG ACG ATA TGG	764
		Antisense: GAT CTT GAT CTT CAT GGT GCT AG	

**Table 2:** Quantitative summary of gross observations in MIA-induced osteoarthritic rats treated with GCSB-5.

		Control	MIA			
			Vehicle	GCSB-5 (mg/kg)		Diclofenac
				300	600	5 mg/kg
Swelling						
No change	0	10/10	0/10	1/10	1/10	0/10
Mild	1	0/10	3/10	8/10	6/10	8/10
Severe	2	0/10	7/10	1/10	3/10	2/10
Average score		0.0 ± 0.0	1.7 ± 0.2^a^	1.0 ± 0.2^a,b^	1.2 ± 0.2^a^	1.2 ± 0.1^a^

Limping						
No change	0	10/10	0/10	6/10	5/10	5/10
Mild	1	0/10	7/10	4/10	5/10	5/10
Severe	2	0/10	3/10	0/10	0/10	0/10
Average score		0.0 ± 0.0	1.3 ± 0.2^a^	0.4 ± 0.2^c^	0.5 ± 0.2^c^	0.5 ± 0.2^c^

GCSB-5 or diclofenac was treated daily for 14 days after 2 weeks of OA induction by intra-articular injection of MIA.

^a^Denotes significant differences (*P* < 0.01) versus the control group.

^b,c^Denote significant differences (*P* < 0.05,   *P* < 0.01) versus the vehicle-treated MIA group.

**Table 3:** Summary of microscopic findings.

		MIA			
		Vehicle	GCSB-5 (mg/kg)		Diclofenac
			300	600	5 mg/kg
Structural changes in the joint					
Surface irregularities	+	0/4	2/4	1/4	2/4
	++	0/4	1/4	2/4	2/4
	+++	4/4	1/4	1/4	0/4
Average pathology score		3	1.8	2	1.5

Ulceration	+	0/4	1/4	3/4	1/4
	++	1/4	1/4	1/4	2/4
	+++	3/4	2/4	0/4	1/4
Average pathology score		2.8	2.3	1.3	2

Fibrillation of cartilage surface	+	0/4	1/4	2/4	3/4
	++	3/4	3/4	1/4	1/4
	+++	1/4	0/4	1/4	0/4
Average pathology score		2.3	1.8	1.8	1.3

Disorganization of chondrocytes	+	0/4	1/4	3/4	3/4
	++	2/4	3/4	1/4	0/4
	+++	2/4	0/4	0/4	1/4
Average pathology score		2.5	1.8	1.3	1.5

Exposure of subchondral bone	+	2/4	1/4	0/4	0/4
	++	1/4	0/4	1/4	0/4
	+++	1/4	0/4	0/4	0/4
Average pathology score		1.8	0.3	0.5	0

Cellular changes of chondrocyte					
hypertrophy	+	1/4	3/4	1/4	0/4
	++	2/4	1/4	3/4	4/4
	+++	1/4	0/4	0/4	0/4
Average pathology score		2	1.3	1.8	2

Degeneration/necrosis	+	0/4	1/4	4/4	2/4
	++	1/4	0/4	0/4	2/4
	+++	3/4	3/4	0/4	0/4
Average pathology score		2.8	2.5	1	1.5

Inflammatory cell infiltration	+	1/4	3/4	2/4	3/4
in synovial tissue	++	1/4	1/4	1/4	0/4
	+++	2/4	0/4	0/4	1/4
Average pathology score		2.3	1.3	1	1.5

Synovial cell proliferation	+	1/4	3/4	3/4	3/4
	++	2/4	0/4	0/4	1/4
	+++	1/4	1/4	1/4	0/4
Average pathology score		2	1.5	1.5	1.3

Safranin-O staining					
Reduction of staining in cartilage	+	0/4	3/4	1/4	1/4
	++	0/4	0/4	0/4	1/4
	+++	4/4	1/4	0/4	0/4
Average pathology score		3	1.5	0.3	0.8

Total pathology score (average ± S.E.M)		24.5 ± 1.3	16.1 ± 1.4^a^	12.5 ± 1.1^b^	13.4 ± 2.2^b^

+: mild, ++: moderate, and +++: severe.

^a,b^Denote significant differences (*P* < 0.05, *P* < 0.01) versus the vehicle-treated MIA group. *N* = 4.

**Table 4:** Effect of GCSB-5 on serum TNF-**α**, IL-1**β*,* and IL-10 levels in MIA-induced osteoarthritic rats.

Group	TNF-**α** (pg/mL)	IL-1*β* (pg/mL)	IL-10 (pg/mL)
Control	30.0 ± 4.5	29.1 ± 3.7	27.8 ± 0.6
MIA			
Vehicle	85.4 ± 6.6^b^	99.5 ± 11.2^b^	60.4 ± 7.2^b^
GCSB-5			
300 mg/kg	60.0 ± 4.6^a,c^	102.6 ± 10.4^b^	91.4 ± 8.8^b,d^
600 mg/kg	55.3 ± 6.2^c^	93.9 ± 9.0^b^	84.5 ± 8.5^b,c^
Diclofenac 5 mg/kg	50.1 ± 4.0^d^	49.5 ± 7.5^d^	86.1 ± 9.2^b,c^

The serum concentration of TNF-**α**, IL-1**β*,* and IL-10 was determined using enzyme-linked immunosorbent assay. The results are presented as mean ± S.E.M. of 6 rats per group.

^a,b^Denote significant differences (*P* < 0.05, *P* < 0.01) compared with control group.

^c,d^Denote significant differences (*P* < 0.05, *P* < 0.01) compared with vehicle-treated MIA group.
